# Relationship of left atrial size and function to invasive left ventricular filling pressure: a cardiac MRI study

**DOI:** 10.1186/1532-429X-14-S1-P233

**Published:** 2012-02-01

**Authors:** Kanna Posina, Jeannette McLaughlin, Peter D Rhee, George A Petrossian, Nathaniel Reichek, Jie J Cao

**Affiliations:** 1 Saint Francis Hospital, Glen Cove, NY, USA

## Background

Increased left atrial (LA) size measured at left ventricular (LV) end systole is associated with cardiovascular morbidity and mortality in population based studies. LA function can be divided into 3 components: LA filling during LV systole, a passive emptying phase during early diastole and active atrial systole during LV late diastole. We sought to assess the relationships of LA volume and emptying function to invasive LV end diastolic pressure (LVEDP).

## Methods

Forty one patients underwent cardiac MRI (CMR) within 5 hours of clinically indicated left heart catheterization. Sixteen healthy volunteers served as CMR controls. LV and LA volumes and ejection (EF) were assessed from cine images. Using the biplane formula, LA volume was calculated at LV end-systole (LAVmax), LV end-diastole (LAVmin) and at the end of LV early filling before LA systole. LA function was divided into global emptying function (LAEF), passive LAEF and active LAEF expressed as fractional volume change. LVEDP was measured invasively during diagnostic catheterization. The association of LA indices with LVEDP was assessed using Receiver Operating Characteristics.

## Results

Mean age was 59 years in patients and 53 years in control (NS). As compared to controls, patients had larger LVEDV (80±15 ml/m2 vs. 90±39 ml/m2, p=0.029) and lower LVEF (57±5% vs. 49±16%, p=0.006). Mean LVEDP was 14±8 mmHg in patients. Of the LA volume indices LAVmin was significantly more closely associated with LVEDP elevation (>12 mmHg) (AUC 0.765, p=0.002) than LAVmax (AUC 0.677, p=0.054) (p=0.048 for LAVmin vs. LAVmax). LAVmin ≥ 23 ml/m2 was associated with LVEDP elevation with 86% sensitivity and 63% specificity. Of the LAEF indices global LAEF had the closest association with LVEDP elevation (AUC 0.780, p<0.001), followed by active LAEF (AUC 0.698, p=0.017) and passive LAEF (AUC 0.646, p=0.099). Global LAEF ≤ 35% was associated with LVEDP elevation with 71% sensitivity and 89% specificity while active LAEF ≤16% was associated with LVEDP elevation with 43% sensitivity and 93% specificity.

## Conclusions

LAV measured at LV end diastole had a stronger association with LVEDP than LAV measured at LV end systole. Global and active LAEF were significantly reduced with increasing LVEDP.

## Funding

None.

